# Heterogeneity of math difficulties and its implications for interventions in multiplication skills

**DOI:** 10.1590/1980-57642018dn12-030006

**Published:** 2018

**Authors:** Mariuche Rodrigues de Almeida Gomides, Gizele Alves Martins, Isabela Starling Alves, Annelise Júlio-Costa, Antônio Jaeger, Vitor Geraldi Haase

**Affiliations:** 1 Graduate Program in Psychology: Cognition and Behavior, Federal University of Minas Gerais, Belo Horizonte, MG, Brazil.; 2 Graduate Program in Neuroscience, Federal University of Minas Gerais, Belo Horizonte, MG, Brazil.; 3 Developmental Neuropsychology Laboratory (LND, UFMG), Belo Horizonte, MG, Brazil.; 4 Department of Educational Psychology, University of Wisconsin-Madison, Madison, WI, USA.; 5 Department of Psychology of the Federal University of Minas Gerais, Belo Horizonte, MG, Brazil.; 6 Instituto Nacional de Ciência e Tecnologia sobre Cognição, Comportamento e Ensino (INCT-ECCE), São Carlos, SP, Brazil.; 7 Graduate Program in Children’s and Adolescents Health, Federal University of Minas Gerais, Belo Horizonte, MG, Brazil.

**Keywords:** multiplication, intervention, learning disabilities, dyscalculia, multiplicação, intervenção, transtornos de aprendizagem, discalculia

## Abstract

Math learning disability (MLD) is a heterogeneous condition characterized by severe and persistent difficulties in learning math, including difficulties in learning multiplication facts.

**Objective:**

In this article, we compared the responses of two MLD children to multiplication facts training.

**Methods:**

One of the children was a 9 year-old girl (HV) who presented mild math difficulties associated with lower accuracy of the Approximate Number System (ANS). The other was an 11 year-old boy (GA) who presented severe math difficulties related to impaired phonological processing due to developmental dyslexia. Both children underwent an intervention for multiplication, comprising conceptual instructions and retrieval practice of the times table.

**Results:**

HV’s accuracy and response speed improved consistently on both training tasks, while GA’s accuracy improved on the Simple Calculation Task only. Error analyses indicated that, after training, HV produced fewer errors of the type “close miss”, and GA produced less omission but more operand errors.

**Conclusion:**

We argue that these differences between their responses to the training tasks were caused by differences in the mechanisms underlying their math difficulties. These results support the notion that individual specificities regarding math disabilities should be taken into account during preparation of training interventions.

## Resumo

O transtorno de aprendizagem da matemática (MLD) é uma condição heterogênea caracterizada por dificuldades acentuadas e persistentes na aprendizagem da matemática, incluindo déficits na aprendizagem dos fatos de multiplicação.

**Objetivo:**

No presente artigo, nos comparamos a resposta de duas crianças com MLD em uma intervenção da multiplicação.

**Métodos:**

Uma das crianças, HV, sexo feminino, 9 anos de idade, apresentava dificuldades menos acentuadas na matemática, associadas a um déficit no Sistema Numérico Aproximado (ANS). A outra criança, GA, sexo masculino, 11 anos de idade, apresentava dificuldades na matemática mais acentuadas associadas a um comprometimento no processamento fonológico devido a um quadro de Dislexia do Desenvolvimento. Ambas as crianças passaram por um programa de intervenção para a multiplicação, o qual se consistia em instruções conceituais e práticas de memorização da tabuada.

**Resultados:**

HV apresentou uma melhora consistente na acurácia e tempo de resposta nas duas medidas de desfecho, enquanto, GA apresentou uma melhora apenas na Tarefa de Cálculos Simples. Análises nos tipos de erros evidenciam que, após a intervenção, HV cometeu menos “erros de aproximação”, ao passo que, GA cometeu menos erros por omissão, mas mais erros de operando.

**Conclusão:**

Nossa hipótese é de que as diferenças na resposta a intervenção dos participantes estão relacionadas a mecanismos subjacentes distintos à aprendizagem da matemática. Esses resultados reforçam a necessidade de que as especificidades nas dificuldades na matemática de cada paciente sejam levadas em consideração durante o planejamento das intervenções.

Math learning disability (MLD) is a disorder characterized by a persistent deficit in mathematical abilities acquisition.^1^^,^ 2 It has a prevalence of 3 to 6% among school-age children,^3^^,^ 4 and is a heterogeneous phenomenon, since it may involve deficits in a variety of cognitive processes underlying arithmetic processing.^5^^,^ 6 Children with MLD have a marked difficulty in establishing reliable associations between problems and solutions, and consequently fail to make a successful transition from using procedural counting strategies to using retrieval-based resolutions.^7^^,^ 8

Learning arithmetic facts depends on nonsymbolic and symbolic processes. Nonsymbolic processes involve the representation of numerosities in an analog fashion (e.g., “■■■■■”; 9), while symbolic processes involve the representation of numbers as verbal codes (e.g. “five”) or visually coded Arabic digits (e.g., “5”; 9). Nonsymbolic processes are important during initial learning of arithmetic facts, since manipulation of quantities is needed for calculation performance.^2^^,^ 10 As children grow older, however, arithmetic facts become permanently stored as a phonological code in memory, and a verbal route to retrieve them is adopted. In this process, symbolic representations become very important, while the reliance on nonsymbolic representations is diminished.^9^ Thus, because arithmetic facts become stored as a phonological code in long-term memory, deficits in symbolic representations can hamper their retrieval.^11^^-^ 13

This notion is supported by prior studies showing that in typical populations, multiplication performance is highly associated with phonological awareness,^14^^,^ 15 and by previous investigations showing that individuals with deficits in phonological processing, as in Development Dyslexia, exhibit difficulties in the retrieval of arithmetic facts.^15^^,^ 16

Because children with MLD often need cognitive or emotional support,^17^^,^ 18 several studies have investigated remediation approaches for MLD.^19^^,^ 20 These studies, however, are highly diverse in terms of the methodologies adopted, which makes it difficult to establish comparisons and generalizations among them.^20^^,^ 21 Regarding multiplication skills specifically, previous interventions studies have demonstrated the efficacy of repeated exposure through practice to achieve automatization.^22^^,^ 23 Additional evidence shows that the integration of conceptual/procedural knowledge and extensive practice leads to long-lasting positive outcomes.^24^^-^ 26 Alternative methods involve mnemonic strategies^27^^,^ 28 and multisensory associations.^21^^,^ 29

Thus, the main goal of the current study was to investigate the efficacy of a multiplication intervention that focused on integrating conceptual/procedural knowledge and extensive practice in MLD. We also aimed to evaluate whether qualitative error analysis in multiplication can be used as an outcome measure of the intervention, in parallel with accuracy and reaction times (RT).^30^^,^ 31 Additionally, we investigated the effects of the intervention for two distinct profiles of MLD.

Due to MLD heterogeneity, case studies can be a useful approach to characterize this disorder. Recently, our research group published a double dissociation study investigating differences in the cognitive profiles of two patients with MLD.^1^ One of the patients, HV, a 9-year-old girl, showed difficulties restricted to an inaccurate Approximated Number System (ANS), which showed no improvement through intervention.^32^ The other patient, GA, an 11-year-old boy, showed difficulties associated with deficits in phonological processing. These two participants took part in individual sessions in a personalized intervention during one semester. A pre and post-test design was adopted and experimental tasks were used to measure the gains of the intervention.

## METHODS

### Participants

Two children with MLD who signed up for a mathematics intervention in a specialized clinic were selected for the study. They had a clinical diagnosis of MLD, according to DSM-IV-TR criteria,^33^ based on their clinical reports and a neuropsychological assessment. The clinical and neuropsychological aspects of both children were reported in previous publications (Haase et al., 2014; Júlio-Costa et al., 2015). HV, a 9-year-old girl enrolled in 3rd grade of a private school, had high intelligence (see 1 for a complete description of HV’s profile), and excellent performance in phonological processing, visuospatial, and executive functions tasks. However, HV scored below average on the nonsymbolic magnitude comparison task and in arithmetic calculations, with substantial impairment in arithmetic facts retrieval. Before the intervention on multiplication, HV took part in an intervention for ANS, which proved unsuccessful.^32^ The present study was conducted in accordance with the procedures required by the Institutional Review Board of the Federal University of Minas Gerais.

The other participant was GA, an 11-year-old boy, who exhibited both MLD and dyslexia. GA was enrolled in the 5^th^ grade of a public school. He had normal intelligence, but difficulties in phonological processing, reading, spelling, motor dexterity, and executive functions. Regarding math, GA exhibited difficulties in verbal aspects, such as number transcoding and word arithmetic problems. Before this intervention, GA participated in a two-year intervention to improve his phonemic awareness and number transcoding abilities (see 1 for a complete description of GA’s profile).

### Intervention

The present intervention in multiplication skills is part of a multi-componential program with 5 independent and semi-hierarchically organized modules,^34^^,^ 35 as proposed by Kaufmann et al.^36^ According to Kaufmann et al.,^36^ mathematical abilities are a modular and hierarchically organized complex domain, in which development of later competencies depend on the acquisition of more basic ones. For instance, children need to master addition before multiplication. In our program, the modules are independently applied according to the profile of each child, since the intervention is personalized. In order to increase children’s motivation and engagement, they do not receive redundant training on skills they have already mastered. The current multiplication training involved two broad strategies: conceptual training and extensive practice.

The conceptual training focused on the understanding of conceptual and procedural contents, and followed principles proposed by Fuchs et al.^37^ The main goal of this strategy was to promote a better comprehension of arithmetic operations and principles, as well as better execution of algorithms and strategies.^38^ On the other hand, the extensive practice training involved repeated production of multiplication facts followed by immediate feedback.^29^ Such practice promotes automatization, and precludes the potential strengthening of incorrect problem-response associations.^39^

### Sessions

Conceptual knowledge was introduced in the first sessions using concrete materials (Golden Bead Material and Montessori blocks). In the training stage, sessions were dedicated to learning multiplication facts from 1 to 9, presented in increasing order of difficulty. The number one times table was introduced first, followed by the facts involving numerals two, five, and nine. The remaining tables (for digits 4, 6, 7, and 8, respectively) were taught in a sequence, with each session of the module dedicated to one of the tables.^22^^,^ 28 A quick review of all the tables that were previously taught during the module was performed at the end of each session. Different types of stimuli were used in the practice problems, auditory and visual. For the auditory stimuli, the therapist read the problems to the child, who gave the answer verbally; whereas for the visual stimuli, the participant was presented with a sheet containing the table problems and wrote down the answer. Recreational materials (e.g., multiplication flash cards: FatFun®) and a token system were used as motivational tools. At the end of every session, the participants won pretend money bills and, at the end of the module, they exchanged these bills for prizes (e.g. books and stickers). Both interventions obeyed the principles described above. Our approach was a series of case studies. Interventions were customized and adapted to the profile, performance level, and main difficulties of each child, and families’ convenience. GA participated in 17 sessions of 90 minutes each, accomplishing about 25 hours of intervention, distributed over a period of 5 months. HV took part in 18 weekly sessions of 60 minutes each, totaling 18 hours of intervention, distributed over a period of 5 months.

### Instruments

*Simple calculation task:* The simple calculation task (SCT) is a paper and pencil task with addition (27 items), subtraction (27 items), and multiplication (28 items) operations. Children were instructed to write the answer for the facts as fast and as accurately as possible. The arithmetic operations included two blocks each with different levels of complexity. Children were initially presented with the addition subtask, followed by the subtraction and multiplication subtasks. Time limit was 60s per blocks. For a complete description of this task, see 1.
*Multiplication table task:* The multiplication table task (MTT) consists of all multiplication facts from 1 to 9. An oral version of the task was administered with GA, while a computerized version of the task was administered for HV. The computerized version was programed with the software Presentation*®*. The task included all possible pairwise combinations of operands from 1 to 9 (81 problems). Ties (e.g., 3×3) were only presented once. Problems were presented in a pseudorandom order. Each trial started with a fixation cross presented for 1s on the center of the computer screen. After fixation, the multiplication problem appeared. Presentation format was horizontal Arabic, colored in white upon a black background. Responses were self-paced and participants were instructed to give oral answers as fast as possible without sacrificing accuracy. Both accuracy and RTs were recorded. In order to record RT, trained examiners pressed the space bar as soon as the participant began to emit a vocal response. Participants were also instructed to avoid producing any kind of vocalization, such as “humm”, before producing the actual responses.

In the oral version of the task, the same stimuli were presented orally by the examiner, one at a time. The examiner recorded RT and accuracy on a sheet of paper. The examiner started recording the time immediately after the problem was read and ended it as soon as the child responded. Problems were presented following a pseudorandom order. Responses were self-paced, and participants were instructed to give oral responses as fast and as accurately as possible.

### Procedures

This study was approved by the local research ethics committee, and the research procedures followed the Helsinki convention.

A within subjects pre- and post-test design was adopted. Potential improvements produced by the intervention were measured by the SCT and the MTT. McNemar tests and Wilcoxon tests were conducted to compare the pre- and post-test scores. For both tests, a significance level of p< .05 was adopted.

Differences in patterns of errors between pre and post-test in the MTT were also examined through a qualitative analysis. Errors were classified into four categories: Operand errors, close-miss errors, table errors and non-table errors.^31^ Operand errors consist of responses that are a multiple for one of the operands (e.g., 5×6=35). In the close-miss errors, the response is within plus or minus ten percent of the correct result (e.g., 4×7=29). Responses that belong to a different table from both operands (e.g., 3×6=20) are considered table errors, and responses that are not present in any multiplication table (e.g. 5×6=59) are categorized as non-table errors. Errors were classified following the procedures proposed by Butterworth et al.^31^

## RESULTS

### HV

Pre- and post-test comparisons of HV’s performance on the SCT showed no significant differences between addition and subtraction blocks. Although she presented higher scores in both multiplication blocks on the posttest relative to pretest, no significant improvement was found. HV’s results are exhibited in Table 1.

**Table 1:** HV’s accuracy on the pretest and post-test for the SCT.

		Pretest	Posttest	McNemar	
		% correct responses		χ^2^	*p*
Addition	1^st^ Block	1.00	.91	< .01	>.05
	2^nd^ Block	.73	.53	1.33	>.05
Subtraction	1^st^ Block	.91	.75	.50	>.05
	2^nd^ Block	.46	.26	1.33	>.05
Multiplication	1^st^ Block	.66	1.00	3.2	>.05
	2^nd^ Block	.15	.46	2.25	>.05

The statistical analyses were performed on the raw data, however, Table 1 shows the percentage of correct responses to facilitate understanding of the data by the reader.

**Figure 1:**
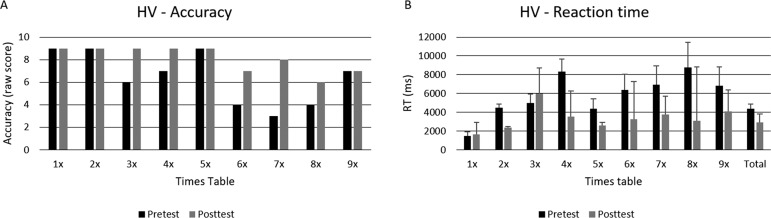
HV’s raw score and median reaction time (ms) in the MTT. [A] shows HV’s raw score related to accuracy at pre- and posttest measures for each times tables. [B] shows HV’s median reaction time (ms) and standard error (represented by the error bars attached to each column) at pre- and posttest measures for each times tables.

HV’s accuracy and RT performance improved on the MTT. HV’s number of correct responses in the multiplication table task increased from 58 to 73 (out of 81), showing significant differences between pre- and post-test, χ^2^=13.06; *p*< .001 (Figure 1A). The RT analysis showed that HV’s responses to multiplications were significantly faster for the posttest, Z= -3.61; *p*< .001. Also, she was faster at solving only small times tables, such as the 2 (Z= -2.10; *p*< .05), 4 (Z= -1.99; *p*< .05) and 5 (Z= -2.42; *p*< .05), but not for larger times tables, highlighting her persistent difficulty in retrieving responses for large multiplication problems (Figure 1B), the same pattern of difficulties was evident on the SCT. However, qualitatively, HV’s accuracy showed an increase in the 6, 7, and 8 times tables in parallel with slower responses (increased RT) to these same tables.

HV’s qualitative error analysis also showed changes in the pattern of errors after the intervention. Most errors committed by HV on pretest were categorized as close-miss errors (10 errors), followed by operand errors (7 errors), non-table (4 errors) and table errors (2 errors). At posttest, there was a decrease in close-miss errors (2 errors), although the number of operand errors (6 errors) remained similar.

### GA

GA’s pre and post-test analysis of the SCT showed a significant performance decrease after the intervention for the small addition set. In the posttest, he was able to solve fewer problems within the time limit, but did not commit errors. In contrast, there was no significant difference between the pre and post-tests for the large addition condition, nor for the small and large subtractions. He had higher scores on both multiplication blocks in the posttest, but a significant accuracy improvement was observed only for small multiplication problems. GA’s results are exhibited in Table 2.

**Table 2:** GA’s accuracy on the pretest and posttest for the SCT.

		Pretest	Posttest	McNemar	
		% correct responses		χ^2^	p
Addition	1^st^ Block	1.00	.50	4.16	<.05
	2^nd^ Block	.53	.60	< .01	>.05
Subtraction	1^st^ Block	.58	.50	< .01	>.05
	2^nd^ Block	.13	.20	< .01	>.05
Multiplication	1^st^ Block	.00	.73	9.09	<.01
	2^nd^ Block	.07	.38	2.25	>.05

The statistical analyses were performed on the raw data, however, Table 2 shows the percentage of correct responses to facilitate understanding of the data by the reader.

**Figure 2:**
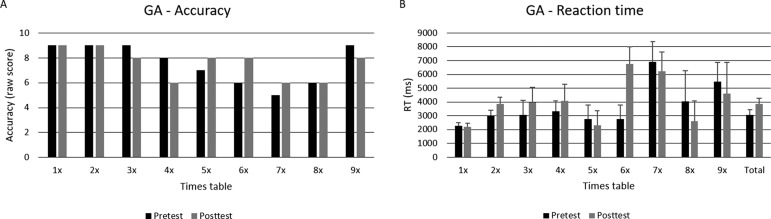
GA’s raw score and median reaction time (ms) in the MTT. [A] shows GA’s raw score related to accuracy at pre- and posttest measures for each times tables. [B] shows GA’s median reaction time (ms) and standard error (represented by the error bars attached to each column) at pre- and posttest measures for each times tables.

GA had the same rates of correct responses on both MTT assessments (i.e., raw scores on pre and post-test of 68 and 69 out of 81 items, respectively) with no significant difference, χ^2^< .01, *p*>.05. He also committed more errors in large multiplication problems, as on the SCT (Figure 2A). The RT analysis revealed that GA was significantly slower at solving the multiplication operations on the posttest than the pretest, Z= -2.06; *p*<.05. No significant differences in RT were observed in the multiplication tables separately (Figure 2B)

The qualitative error analysis revealed a change in the error pattern. The errors most frequently presented by GA’s on the pretest were from the omission category (12 errors). He was unable to respond through memory retrieval, and failed to employ procedural strategies. One additional error was classified as an operand error in the pretest. At posttest, a decrease in omission errors (6 errors) accompanied by an increase in systematic errors, such as operand errors (5 errors), was observed. In addition, one error was classified as a non-table error.

Overall, HV showed enhancement in accuracy, response speed, and strategy in response to training. On the other hand, despite the strategy shifting and gains obtained in accuracy on the SCT, GA’s response speed worsened.

## DISCUSSION

In this study, we investigated the efficacy of an intervention program on multiplication facts in two children with MLD: GA, a boy with MLD and deficit in phonological processing , and HV, a girl with severe persistent deficit in the ANS.^1^ We adopted a within-subjects design comparing each child’s response to intervention against their own baseline. At the end of the intervention, an improvement in HV multiplication performance was observed for accuracy and RT. This improvement, however, was not evident for GA. In this section, we discuss the results of the intervention for HV and GA, and contrast their profiles.

Indeed, a poorer RT performance was exhibited by GA after the intervention. Importantly, GA’s results on the multiplication blocks of the SCT were inconsistent with the results found on the MTT. GA showed an accuracy improvement in the small multiplication block of the SCT, but a worse RT performance for the MTT. On the other hand, the qualitative error analysis suggested a shift in the error pattern from non-systematic errors to systematic errors in both patients after the end of the intervention.

GA’s outcomes regarding multiplication performance were inconsistent. Two hypotheses could potentially account for GA’s results. First, the results can be interpreted according to differences in stimuli presentation between participants. In contrast to HV, GA responded to an oral version of the MTT, while for the SCT, stimuli were presented in arabic-visual form to both patients. As he exhibited phonological processing deficits, stimuli presented verbally could be more difficult for him than arabic-visual presentation. Hence, the MTT may not have been suitable to evaluate GA’s improvement in multiplication. Alternatively, while in the MTT there was no time limit to finish the task, an interruption criterion of 60 s per block was adopted for the SCT. Since GA employed immature strategies, such as finger counting, his performance at pretest may have been impaired by the limited time available to produce a response. At the end of the intervention, he was able to use more efficient strategies, showing an improvement at posttest. It is important to point out, however, that these hypotheses do not exclude one another.

The change in the error pattern from non-systematic to systematic errors may reflect a reorganization of the arithmetic facts network in both cases.^26^ In addition, the shift from non-systematic to systematic errors has been considered a positive effect of multiplication facts interventions in patients with acalculia due to an aphasic condition.^25^^,^ 26 However, these effects in children with MLD have yet to be investigated.

Type of error can be an important clue towards understanding the strategies used by patients to solve arithmetic problems. Close-miss was the most common error committed by HV in the pretest. She used finger counting as a compensatory method for her automatization difficulties. As finger counting is an error-prone strategy, HV may have committed mistakes during counting procedures. In addition, failures in backup-strategy implementation may lead to false associations in memory.^39^ In contrast, the number of omission errors observed at GA’s pretest was associated with his difficulties employing procedural strategies to compensate for retrieval problems.

As predicted, children with distinct cognitive profiles in relation to MLD can respond differently to similar intervention programs. GA had a math difficulty pattern related to verbal deficits. Multiplication can be considered a verbal domain competence since it is assumed to rely on verbal codes. Several studies have reviewed the association between multiplication and verbal skills (see 16 for a review). For example, Hecht et al.,^40^ in a longitudinal study, showed that phonemic processing skills assessed at 2^nd^ grade were predictive of school performance in mathematics until the 5^th^ grade.

HV’s difficulties, on the other hand, seem to be restricted to an inaccurate ANS. McCrink and Spelke^41^ investigated the relationship between the ANS and the intuitive process of multiplication. They showed that 5 to 7-year-old children, who were not formally introduced to multiplication, could understand nonsymbolic multiplicative patterns, and that this ability relies on the ANS. However, studies with adults suggest that multiplication depends on symbolic-knowledge.^42^ One possibility is that the ANS is important for an initial understanding of the multiplicative property, and that a verbal memory route becomes the main path to resolve multiplication facts through development.

As MLD constitute a heterogeneous group from a neurocognitive point of view,^6^ case studies may be a useful approach to characterize the cognitive deficits underlying developmental disorders. We observed that individuals with distinct MLD profiles responded differently to specific interventions. Therefore, it can be inferred that different cognitive mechanisms are behind their math difficulties. In this sense, the existence of different subtypes of MLD is supported.

The child with verbal deficits had a less substantial improvement after an intervention on multiplication focused on concepts and procedure exposition, and automatization by verbal strategies. As we discussed two cases, with different cognitive-neuropsychological patterns of assets and deficits, interpretation of the findings is necessarily tentative. One hypothesis is that children with verbal difficulties may benefit more from other kinds of interventions based on compensatory methods, such as mnemonics and somatosensory strategies.^21^^,^ 28 In contrast, this program could benefit children with ANS deficits associated with multiplication retrieval difficulties. It is also important to keep in mind that the difficulties of a considerable group of children with MLD are substantial and relatively resistant to intervention.^43^^,^ 44 Despite the effects of the interventions, the current study has some limitations. Further studies should investigate the program applicability to larger samples, as well as to other cognitive profiles of impairments. Long-term effect should also be addressed. As the present study used a clinical approach, experimental studies with stricter control of variables are also important.
